# Exploring the Pain Situation, Pain Impact, and Educational Preferences of Pain Among Adults in Mainland China, a Cross-Sectional Study

**DOI:** 10.3390/healthcare13030289

**Published:** 2025-01-31

**Authors:** Jiafan He, Mimi Mun Yee Tse, Tyrone Tai On Kwok, Timothy Chung Ming Wu, Shukkwan Tang

**Affiliations:** 1 School of Nursing and Health Sciences, Hong Kong Metropolitan University, Hong Kong 999077, Hong Kong; mmytse@hkmu.edu.hk (M.M.Y.T.); ttokwok@hkmu.edu.hk (T.T.O.K.); tcmwu@hkmu.edu.hk (T.C.M.W.); 2 School of Nursing, Caritas Medical Centre, Hospital Authority, Hong Kong 999077, Hong Kong

**Keywords:** educational preferences, functional limitation, pain, pain management, mainland China

## Abstract

Objectives: This study aimed to investigate the pain situation, functional limitations, treatment used, care-seeking behaviors, and educational preferences of adults with pain in mainland China. Methods: An online questionnaire was developed through expert validation, and participants were recruited via social media platforms. Inclusion criteria required having access to the Internet and smartphones, while individuals with significant cognitive impairments or severe mental illness were excluded. Results: 1566 participants, predominantly male (951) with a mean age of 30.24, were included. A total of 80.1% of the respondents reported experiencing pain, with over half suffering from chronic pain. Pain primarily affects the neck, lower back, and upper back, especially chronic low back pain. Pain significantly impacted various aspects of life, including mood, physical activity, work performance, family dynamics, and social relationships, particularly among chronic pain sufferers (*p*-value: < 0.001). Analgesics (66.9%) and self-management (80–94.3%) were the most used pain management strategies, with respondents with chronic pain reporting higher usage and effectiveness of medication than those with acute pain (*p*-value: < 0.001). Participants also expressed a greater interest in online education and psychotherapy interventions, especially through mobile applications. Conclusions: Chronic pain is highly prevalent in mainland China, leading to emotional distress, decreased work competency, and social isolation, with a strong demand for pain education through smartphone applications.

## 1. Introduction

The Global Study on Disease Burden highlights the substantial global prevalence and impact of pain [1], manifested as persistent discomfort in muscles, bones, joints, or tendons [2]. Acute pain refers to pain persisting for less than 3 months, with chronic pain lasting for more than 3 months with non-cancer pain [3]. The advent of the COVID-19 pandemic exacerbated pain experiences, with adults reporting deteriorated pain and post-pandemic health status compared to pre-pandemic levels, especially in patients seeking clinical care [4]. Addressing pain would be beneficial in helping this population maintain a productive life.

In China, several studies have revealed that the prevalence of pain was 57.3% among young adults and 45% among adults [5,6], and most individuals reported a “very slight” level of pain intensity [7]. Individuals with pain face not only the risk of enduring pain symptoms but also contend with restricted social function, impeding daily living and work [8,9]. A prospective study also found the negative impacts of pain on work, social life, and family responsibility in mainland China, Taiwan, and Hong Kong [10]. However, despite the functional limitations of pain, 24.1% of Chinese patients did not seek medical help, and 36.79% of patients never received any treatment because they believed chronic pain is not harmful [11]. As the capacity to perform daily life activities and effectiveness in pain reduction are regarded as important attributes in patient preferences for the treatment of chronic musculoskeletal pain [12], and as previous studies have only focused on a specific age group or used snowball sampling without the substantial recruitment of participants, a thorough understanding of the current scope of pain in mainland China and the differences between acute and chronic pain is needed for appropriate treatment.

Pain education (PE) has shown significant efficacy in various painful conditions, leading to reduced fear, improved patient perceptions of pain, and immediate enhancements in attitudes toward pain [13]. Martorella (2017) highlighted that interventions should be aligned with patient-specific requirements [14]. However, the extent to which measures are informed by prior assessments of a patient’s needs and preferences remains unclear [15,16]. Differences in perceptions between healthcare professionals and patients regarding pain management are well documented [17], influenced by cultural factors that shape pain prevalence, coping mechanisms, and beliefs [18,19]. Understanding the information regarding patients’ seeking behaviors related to pain and their preferred methods for pain management is crucial for enhancing the effectiveness and applicability of pain education. However, in mainland China, preferences regarding the delivery method and the nature of pain education remain unclear.

While previous studies have typically concentrated on specific age groups or localized regions, this study aimed to provide a national perspective on pain prevalence, functional limitations, and treatment-seeking behaviors across diverse demographics. It also explored the educational preferences of pain sufferers, an area that has received limited attention in the context of mainland China, particularly regarding online self-management methods. While much research has focused on the negative impacts of pain, this study filled a significant gap by investigating the differences between acute and chronic pain.

The study aimed to investigate the pain situation, functional limitations, treatment used, care-seeking behaviors, and educational preferences of adult populations in mainland China. The objectives of the present study were to (1) investigate the pain situation, functional limitations, treatment preference, and care-seeking behaviors among adults in mainland China; (2) explore the differences in terms of acute and chronic pain; and (3) determine the educational needs and preferences of adult populations with pain in mainland China.

## 2. Materials and Methods

### 2.1. Study Design and Participants

This cross-sectional study was conducted from December 2023 to March 2024 in mainland China. Data collection was conducted through an online self-administered questionnaire. Participants were recruited through social media platforms such as WeChat, Weibo, and Wenjuanxing (www.wjx.cn, accessed on 30 December 2023) [20]. The sample size was computed using the formula N = Zα2P (1 − P)/d2 [21], where Zα = 1.96 is the standard normal variate at 5% type 1 error (i.e., α = 0.05). The estimated acceptable margin of error for proportion d was 0.03, and the prevalence (P) of respondents with pain was estimated at 45% [6]. Finally, the minimum sample size was estimated at around 1100, and the power was 0.95.

The inclusion criteria were as follows: (1) individuals aged between 18 and 60 years; (2) residents of mainland China proficient in Chinese; and (3) owners of smartphones with Internet access. Exclusion criteria included individuals with drug addiction, dyslexia, intellectual disability, or severe mental illness. The location was recorded using participants’ smartphone GPS receivers. To avoid duplication of data, each internet network IP address was granted access once only to complete the questionnaire. A total of 1623 questionnaires were distributed, 1618 completed responses were returned, and then 52 responses were discarded according to the exclusion criteria—equating to a 96.5% usable response rate.

### 2.2. Measures

A self-administered online-based questionnaire was developed based on a study by Tang et al. (2021) [22]. Initially, the questionnaire was translated into Mandarin Chinese to ensure acceptability and comprehensibility, following which modifications were made to the translated text to align it with Chinese culture. Four items, including the functional limitation of pain at work, socially, and when with family, care-seeking behaviors, and additional intervention needs, were included. Considering the characteristics of Internet usage in mainland China [23], additional items pertaining to patient preferences for pain education methods were included. Furthermore, items assessing the self-assessment of pain and the transition from acute to chronic pain were included to provide a more comprehensive understanding of individual health-seeking behaviors regarding pain management.

The modified questionnaire was validated through a rigorous validation process involving an expert panel of three professionals: two occupational therapists with over 8 years of experience and a clinician specializing in pain management with over 20 years of practice. These experts assessed the content validity of each item using a four-point Likert scale, where the relevance of each item was rated from “not relevant” to “highly relevant”. To ensure the content validity of the questionnaire, the Content Validity Index (CVI) was used, yielding a score of 0.99, which indicated satisfactory content validity [24]. In terms of reliability, a test-retest reliability evaluation was conducted with nine extra participants who completed the questionnaire twice—once at baseline and again 14 days later. The intra-class correlation coefficient (ICC) for the test-retest reliability was 0.643, which was considered to be fair and indicated moderate consistency between the two assessments [25]. Additionally, the measurement tool demonstrated high internal consistency with a Cronbach’s α of 0.92, confirming the reliability of the instrument for this study [24].

The questionnaire comprised five sections that required participants to indicate whether they were experiencing pain. Affirmative responses prompted continuation, whereas negative responses directed participants to the section on sources on pain management education. The first part explored pain history, including duration, site, and intensity. The second section addressed treatment methods and the impact of pain on various aspects of life by asking to what extent the pain had improved due to the treatment on a four-point scale and to what extent pain affected different aspects of life on a four-point scale (0 points: None; ‒3 points: Often). The third section assessed the participants’ past pain management education. Participants were asked to explain whether and where they received pain education, their interest and willingness to participate in pain education, as well as expected content, methods, and settings for pain education. In the fourth section, where the participants obtained pain treatment information, the use of the Internet for pain management was examined as the index of measuring care-seeking behaviors. The last section of this questionnaire included social-demographic data involving age, sex, educational level, marital status, occupation type, resident area, and health status.

### 2.3. Ethical Consideration

This study was approved by the Research Ethics Committee of Hong Kong Metropolitan University (issue no: HE-SF2023/41). Online Informed Consented and Privacy Protection Terms were displayed on the head page of the online questionnaire. Participants could answer questions if they confirmed consent. Participation in this study was completely anonymous, and no personal privacy information was required to be provided in the questionnaire.

### 2.4. Data Analysis

Data analysis was performed using IBM SPSS Statistics for Windows, version 25.0. Cases (rows) with missing data for more than 10 items were excluded from the analysis to ensure the completeness of the data. Descriptive statistics (e.g., frequency, percentage, mean, and standard deviation) were employed to present the prevalence of pain, treatment utilization, and preferences for pain education themes and formats. To analyze univariate relations and adjust for multiple comparisons, chi-square tests and two-tailed t-tests were employed. The Mann–Whitney U-test was used to compare the efficacy of treatment and effects of pain between the acute and chronic pain groups, based on the nature of the data. The statistical significance was set at a *p*-value of <0.05 to ensure that the results were statistically meaningful.

## 3. Results

During the three-month data collection period, a total of 1566 participants met the inclusion criteria, 951 males and 615 females, with a mean age of 30.24. A total of 57 participants who reported excluded criteria, including an unwillingness to continue, being within the wrong age range, and multiple answers from the same IP address, were deleted. The majority (53.8%) of participants were married. The largest groups of participants were professionals (29.1%), clerical support workers (25.0%), and service and sale workers (15.2%). Furthermore, bachelor’s degrees (63.1%) accounted for the majority of participants’ education. Geographically, the participants were from various regions across the country, mainly Central China (25.2%), South China (20.3%), and North China (18.6%). Of the 1566 participants, 501 (32.0%) reported a monthly income between 6001 and 10,000. Most participants (39.7%) reported no history of illness. Of the participants, 80.1% (*n* = 1255) reported the presence of pain, with over half (69.6%) indicating chronic pain (Table 1).

### 3.1. Pain Situations

Of the 1255 participants with pain, 30.4% of them (*n* = 381) experienced acute pain and 69.6% (*n* = 873) had chronic pain. Sex, marital status, occupation, education, residence, and income were different. Compared with the female group, a significantly higher proportion of male participants reported both acute and chronic pain (*p*-value: <0.001). Singles reported more acute pain, whereas those with chronic pain tended to be married (*p*-value: <0.01). A significantly higher percentage of acute pain cases was observed among clerical support workers, while chronic pain was more prevalent among professionals (*p*-value: <0.01). Participants with lower-than-senior secondary education levels exhibited a higher proportion of acute pain, whereas those at other education levels were associated with chronic pain (*p*-value: <0.001). A larger proportion of participants from Central China reported pain than those from North China (*p*-value: <0.01). Participants with a monthly income ranging from 300,001 to 40,000 reported a significantly higher percentage of pain experience (*p*-value: <0.001) (Table 1).

**Figure 1:**
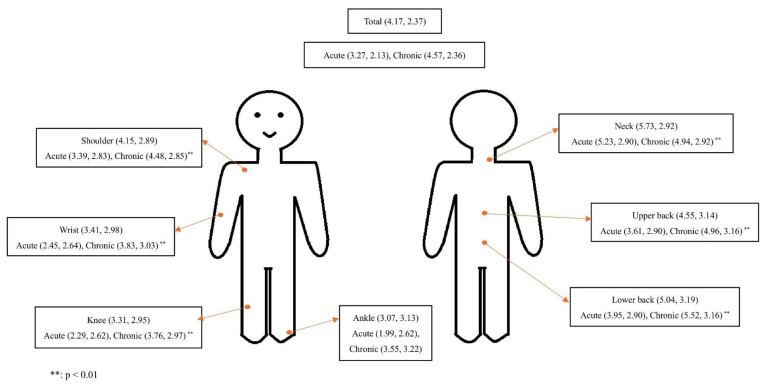
Mean pain intensity and locations.

The total mean pain score of all the pain participants was 4.17 (SD = 2.37), with acute pain registering a mean score of 3.49 (SD = 2.19) and chronic pain registering a mean score of 4.83 (SD = 2.53). Notably, neck, lower back, and upper back emerged as the most common sites of pain, with mean scores of 5.73 (SD = 2.92), 5.04 (SD = 3.19), and 4.55 (SD = 3.14), respectively. In the comparison of mean pain intensity between acute and chronic pain, significantly higher pain intensity was found in the acute pain group with neck pain, whereas other body sites showed significantly higher intensity in the chronic pain group (*p*-value: <0.001) (Figure 1).

### 3.2. Psychological Impact of Pain

**Figure 2:**
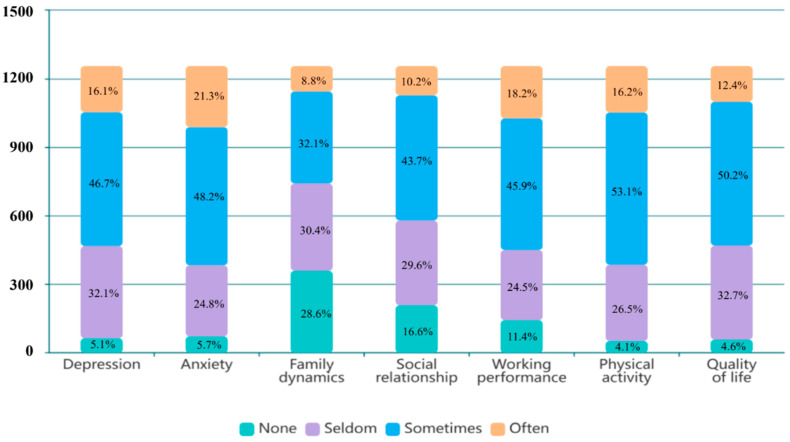
Life impacts of pain.

Participants reported that their pain affected various aspects of psychosocial functions, including mood, family dynamics, and social relationships. Over 90% indicated experiencing distressed moods seldom to often, with sometimes being the most frequently reported frequency. Additionally, 71.4% and 83.4% of participants reported that their pain had varying degrees of impact on their family and social aspects (Figure 2). Table 2 presents functional limitations: 44.1% of participants reported that their family members were unable to understand their pain suffering, and 37.7% reported an inability to do housework. More than half of the participants expressed fear of experiencing sudden pain while meeting others, and 47.7% reported unwillingness to share their pain experiences.

Comparing different pain groups, participants with chronic pain exhibited significantly higher levels of disturbances in mood, family dynamics, and social relationships (*p*-value: < 0.001). Participants with chronic pain also indicated that pain posed a moderate risk factor in their ability to care for family members and perform household chores (*p*-value: < 0.05), with 47.2% reporting that their families could not understand their suffering (*p*-value: < 0.01). Regarding impact on social life, participants with chronic pain reported a significantly higher ratio of social isolation, including unwillingness to meet friends (35.6%), reluctance to share their pain experiences (52.2%), and fear of experiencing sudden pain during social interactions (55.4%) (*p*-value: < 0.001) (Table 2).

### 3.3. Physical and Functional Impact of Pain

Our study revealed that pain exerts a negative impact on work performance, physical activity, and overall quality of life. A substantial majority (88.6%) of the participants reported that pain affected their work performance, with nearly 20% indicating a frequent occurrence. Table 2 illustrates that over 50% of participants experienced difficulties concentrating on work due to pain, while 48.9% reported procrastinating at work because of frequent hospital visits. Moreover, over 90% of the participants reported reduced physical activity and diminished quality of life, with frequencies ranging from seldom to often.

Participants experiencing chronic pain demonstrated significantly higher levels of disruption in work and engagement in physical activities than those with acute pain (*p*-value: < 0.001). Among participants with acute pain, difficulties in concentrating on work due to pain were more prevalent (54.7%) (*p*-value: < 0.001). Conversely, the percentages of work procrastination (53.4%), absenteeism (38.8%), and diminished work competence (39.4%) were significantly higher in the chronic pain group than in the acute pain group (*p*-value: < 0.001).

### 3.4. Perceived Effectiveness of Pain Treatment and Care-Seeking Behaviours

Over 60% of the participants experiencing pain reported using analgesics to manage their symptoms, with the majority (61.0%) perceiving these drugs as effective. Additionally, a substantial proportion of participants indicated resorting to bed rest (94.3%), massage (92.9%), and deep breathing (86.8%) as pain management strategies. However, psychotherapy, nerve stimulation therapy (NST), and aromatherapy were among the least utilized approaches, with adoption rates of 67.8%, 67.2%, and 63.3%, respectively. Regarding the differences between the acute and chronic pain groups, a significantly higher percentage of participants with chronic pain (73.4%) used analgesics compared to participants with acute pain (52.8%) (*p*-value: < 0.001), and participants with chronic pain perceived analgesics as more effective in reducing pain (*p*-value: < 0.001). In addition, recreation, NST, acupuncture, and psychotherapy were reported to be significantly more effective for chronic pain management than for acute pain (*p*-value: < 0.01). While aromatherapy, analgesic balm/oil, and exercise showed moderate effectiveness in participants with chronic pain compared to the acute pain group (*p*-value: < 0.05) (Table 3).

Furthermore, the findings presented in Table 4 reveal that 54.5% and 49.2% of participants expressed an intention to seek pain management through mobile applications and websites, respectively, followed by 47.2% who intended to seek assistance from medical staff. However, 24.2% of participants indicated a lack of resources for pain management. As for the usage of the Internet for pain management, 63.1% of participants reported attempting to search for pain treatments, with 53.7% focusing on chronic pain-related information. However, less than 20% of participants attempted to search for contact with support groups or relaxation interventions. Additionally, the chronic pain group was significantly more active in the seeking support group, the fellow patients’ group, and treatments compared to the acute pain group (*p*-value: < 0.01).

### 3.5. Pain Education Preferences

Over 95% of the participants had never been enrolled in a pain education program before, yet 93.4% expressed interest in participating in one. Specifically, 50.6% found that using mHealth apps to deliver pain education was acceptable, while 64.1% of chronic pain participants expressed a desire to connect with others experiencing similar pain. The settings of the education program were favored for a duration of 2–4 weeks (42.7%) and two to three sessions a week, 30 min each (29.5%). The participants expressed a particular interest in topics such as the effects of pain (60.9%), medications and their side effects (57.1%), self-assessments of pain (52.3%), and understanding how acute pain transitions to chronic pain (50.2%). Beyond pain education, psychotherapy (61.9%) and exercise therapy (56.7%) were the most desired interventions for inclusion in the program.

Regarding the differences between pain groups, chronic pain participants indicated a greater need for fellow patient groups and online education led by intervention deliverers (*p*-value: < 0.05). As for topics, acute pain participants showed a significant interest in the definition and mechanisms of pain, while the chronic pain group favored discussions on the effects of pain, medications, and their side effects (*p*-value: < 0.05). Additionally, the chronic pain group indicated a significantly greater need for psychological interventions (*p*-value: < 0.001) (Table 5).

## 4. Discussion

This study was able to recruit a substantial sample size of 1566 participants, predominantly male (60.7%), to delve into the pain situation, impact, treatment utilization, care-seeking behaviors, and educational preferences among adult populations in mainland China. A high prevalence of pain (80.1%) and chronic pain (69.6%) was observed. Participants with chronic pain reported significantly higher mean pain scores than those with acute pain. Participants with chronic pain exhibited more severe disruptions in their physical and psychological outcomes compared to those with acute pain. However, the study revealed that more than 95% of participants had never received pain education, emphasizing the importance of addressing this gap to improve their quality of life.

### 4.1. Pain Situations

The results of the present study revealed a high prevalence of pain (80.1%) among adults in mainland China. Chronic pain affects a significant proportion (69.6%), surpassing the previous estimates reported in the studies conducted in 2019 (45%) and 2023 (62.2%) [6,26]. The higher prevalence may be due to our sample mostly consisting of individuals with higher education and those from economically prosperous regions, particularly in Central and Southern China, where smartphone ownership is more common. Additionally, people who have experienced pain are more likely to complete the survey, which could lead to an overestimation of pain prevalence. This prevalence exceeds that reported in other Asian regions, including Japan (39.0%) and Korea (45%) [27,28]. Additionally, our analysis underscored the highest intensity of neck, lower back, and upper back pain, followed closely by shoulder pain, aligning with global patterns, which identified low back pain as the leading contributor to years lived with disability, with neck pain also ranking prominently [29]. Differences in pain intensity between the acute and chronic pain groups were observed, with chronic pain exhibiting higher levels across all body sites except for neck pain. A previous study indicated that, in contrast to the recommendations of non-pharmacological intervention for low back pain, the neck pain guidelines included the use of painkillers for acute pain due to spontaneous resolution [30,31].

### 4.2. Pain Impacts

Individuals with pain reported heightened challenges in engaging in physical activities, maintaining social and familial connections, and fulfilling work obligations. Notably, chronic pain sufferers exhibited a greater tendency towards work-related procrastination (53.4% vs. 38.7%), absenteeism (38.8% vs. 30.9%), and diminished work competence (39.4% vs. 35.9%) than those with acute pain, a trend consistent with prior research linking chronic pain to impaired work performance and increased absenteeism [9,32]. Moreover, participants with chronic pain highlighted significant social and familial repercussions, including discordant perceptions of their suffering within their family circles, unwillingness to bring burden to their family, challenges in planning social engagements due to pain unpredictability, and experiences of social isolation from the stigma of chronic pain. These findings are in line with the existing literature [33,34], and they also reflect cultural attitudes in Chinese society where tolerating pain and underreporting suffering are often seen as virtues to avoid burdening others, particularly family members [35]. These findings underscore the importance of managing and treating patients suffering from chronic pain and highlight the deleterious effects of chronic pain on global quality of life.

### 4.3. Perceived Effectiveness of Pain Treatment and Care-Seeking Behaviours

Pain management requires a multimodal approach. Our findings revealed that most patients, particularly those with chronic pain, relied on medication for pain relief. This result demonstrated contrary evidence of frequently negative views regarding opioids [36] but showed consistency with the high incidence of opioids administered at doses exceeding prescriptions in China [37], suggesting a crisis trend of potential opioid dependence in the Chinese population. However, the guidelines advise against medication use due to the heightened risk of adverse events associated with long-term opioid use [38,39]. Additionally, self-management strategies, such as bed rest and distraction, have been commonly reported, indicating limited access to pain management services and lower engagement in pain management in mainland China [35]. Insights from a cultural study shed light on the underlying reasons why Chinese individuals exhibit lower levels of trust in healthcare providers and often defer to the opinions of family and friends in evaluating illnesses [40], which is also consistent with our analysis that a considerable number of participants seek help from e-learning (54.5%) and friends (39.2%). Pain education has been shown to improve pain outcomes [41], yet our study revealed low prior exposure to pain education, highlighting a significant opportunity for improvement in introducing educational interventions to pain management.

### 4.4. Pain Education Preferences

Treatment guidelines increasingly advocate patient involvement in healthcare decision-making, encompassing the relative desirability or acceptability of various treatment options or attributes among patients [42]. This study indicated widespread acceptance of pain education among participants (93.4%), with self-management via e-learning emerging as the preferred modality for adults. This preference may be attributed to a tendency among Chinese to underreport pain perception to reduce the emotional burden on caregivers, a behavior shaped by cultural values that prioritize family harmony and minimize social burden [43]. Additionally, the higher prevalence of smartphone ownership among participants with higher income may lead to an overestimation of demand for e-learning, as those with smartphones are more likely to engage with digital education. The preferred duration and frequency of PE sessions align with existing recommendations, with 4–12 sessions lasting 30 min each [44]. The study also revealed a significant desire for peer support groups among participants with chronic pain, likely due to the social isolation associated with chronic pain [45], particularly for individuals influenced by Confucian values [46] who may experience heightened impairment.

Furthermore, participants identified the effects of pain, the use of painkillers, and self-assessment of pain as the most sought-after topics. It is necessary to understand in what way pain impacts daily functioning, focusing on validating pain, including “my pain is real, acknowledge me, and my feelings; impact of pain on my whole self” [47], because “validation (of pain and self) and reconnection (with self and others) empowers a person to begin a healing journey” [48]. Notably, the assessment of pain intensity is a cornerstone of effective pain management, yet alarmingly there are disparities between patient and physician pain ratings, with patient pain often being underestimated in clinical settings [49]. Asians are less likely to express their pain symptoms to others [50], and they may rely more on self-assessment. This underscores the importance of fostering open expressions of pain and enhancing pain assessment outreach efforts. Guideline awareness has been shown to bolster confidence in managing chronic non-cancer pain [51], given the preference on the topic of medication use in this study (57.1%), and highlight the importance of including information on medication use in pain education.

Gamified education, the inclusion of game elements in non-game contexts, which often incorporates elements such as points, leaderboards, and badges, has emerged as a promising approach by which to enhance patient engagement and connection in pain education [52], especially for Chinese adults, who prefer to self-management. Playing games can distract patients from their fear of pain and the pain itself, a concept that has been examined in previous studies [53]. Given these benefits, there is a pressing need to incorporate gamification into the delivery of pain education for chronic pain management in mainland China.

While many studies have examined pain prevalence and its impact on quality of life [6,26], most of the existing research primarily focuses on chronic pain. This study, however, identifies significant differences in pain prevalence between acute and chronic pain across various body sites. Specifically, we found that chronic pain sufferers experienced higher pain intensity in the lower back and upper back, whereas acute pain sufferers reported significantly higher pain intensity in the neck region. Additionally, while the existing literature has often emphasized the biopsychosocial comorbidities associated with chronic pain [54], our study provides new insights into how acute and chronic pain sufferers experience pain differently in terms of psychological, social, and functional impacts. We also extensively explored the impact of pain on individuals’ ability, and that chronic pain sufferers reported greater feelings of guilt and a lack of empathy from others, while acute pain sufferers were more likely to struggle with work concentration. Moreover, our study emphasizes the role of online self-management as an increasingly accessible method for managing pain, underscoring the potential of digital health interventions for those with chronic pain.

Although we provide some of the first data on education preferences reported directly by pain participants, our study is not without limitations. Since smartphone access is not universally available across all regions of mainland China, this study may have disproportionately represented individuals from higher socio-economic backgrounds who are more likely to own smartphones. This selection bias may skew the study’s conclusions, particularly concerning pain education preferences through mobile applications. Furthermore, the recruitment was distributed through online platforms and employed explicit references to “pain” in the study title, likely attracting individuals who were experiencing pain or were predisposed to pain-related concerns. This self-selection bias may have inflated the pain prevalence in the study sample, compromising the representativeness of the findings, as it may not fully reflect the pain experiences of all adults in mainland China. Additionally, neglecting to examine factors such as gender, educational background, geographic location, or other socioeconomic status may have obscured important variations in pain experiences and perceptions among diverse population groups. Finally, the features in the study only involved biological attributes rather than gender-diverse populations, which neglected potential social aspects of diverse populations. In light of these limitations, future research should aim to implement a more randomized sampling strategy that includes participants from diverse demographic backgrounds, regions, and technological access capabilities, particularly those without smartphone access, to better assess pain education preferences. Additionally, longitudinal studies are needed to monitor how pain prevalence, management strategies, and educational preferences evolve over time, which would provide deeper insights into the long-term dynamics of pain and its impact on individuals. Finally, integrating gamification into pain education may work best for digitally connected individuals, but its broader application may need to be adapted for underserved populations.

Owing to the lack of trust in clinicians from the Chinese population, developing pain education for healthcare professionals is crucial to address the lack of trust in medical practitioners. This includes transitioning towards a patient-centered approach and fostering empathy among healthcare providers. Extending pain education to patient families and the public is also necessary to encourage timely help-seeking behaviors among patients. Additionally, pain evaluation should encompass various aspects, such as pain descriptions, psychological status, and treatment resources, to develop comprehensive treatment plans that could also be included in pain education programs. Furthermore, the development and investigation of gamified pain education approaches based on Chinese culture and using Chinese social media platforms may enhance patient engagement and persistence, as well as help alleviate feelings of loneliness and social isolation among pain patients. In light of these limitations, future research should aim to implement a more randomized sampling strategy that includes participants from diverse demographic backgrounds, regions, and technological access capabilities.

## 5. Conclusions

Pain is prevalent in mainland China. Our study suggested that almost 70% of respondents were suffering from chronic pain. Although various non-pharmacological interventions are widely available, the majority of Chinese adults prefer medications and self-management. Pain can cause a myriad of negative impacts on the various aspects of daily life, particularly for individuals with chronic pain, who experience heightened emotional distress, decreased work competency, and social isolation. There is a strong demand for pain education, especially through smartphone applications for individuals with smartphones that cover pain impact, medication use, self-assessment of pain, and peer support groups. Future research could incorporate sociodemographic factors, such as gender and age, which could provide valuable insights into how these variables influence pain perception and treatment preferences.

## Figures and Tables

**Table 1:** Demographic characteristics of the participants.

	Total (*n* = 1566)		Non-Pain Group (*n* = 311, 19.9%)		Pain Group						*p*-Values ^a^
					Total (*n* = 1255, 80.1%)		Acute Pain (*n* = 381, 30.4%)		Chronic Pain (*n* = 873, 69.6%)		
	*n*	%	*n*	%	*n*	%	*n*	%	*n*	%	
Gender											0.004
Male	951	60.7	182	11.6%	769	49.1%	211	13.5%	558	35.6%	
Female	615	39.3	129	8.2%	486	31.0%	171	10.9%	315	20.1%	
Age											
Mean age	30.24 (9.20)		30.37 (10.59)		30.21 (8.81)		29.50 (8.17)		30.52 (9.06)		0.170
Marital status											0.000
Single	697	44.5	175	11.2%	522	33.3%	199	12.7%	323	20.6%	
Married	843	53.8	130	8.3%	713	45.5%	177	11.3%	536	34.2%	
Divorced/Widowed	26	1.7	6	0.4%	20	1.3%	6	0.4%	14	0.9%	
Occupation type											0.001
Managers and administrators	88	5.6	18	1.1%	70	4.5%	27	1.7%	43	2.7%	
Professionals	455	29.1	89	5.7%	366	23.4%	79	5.0%	287	18.3%	
Clerical support workers	391	25.0	73	4.7%	318	20.3%	114	7.3%	204	13.0%	
Service and sale workers	238	15.2	46	2.9%	192	12.3%	52	3.3%	140	8.9%	
Craft and related workers	96	6.1	22	1.4%	74	4.7%	28	1.8%	46	2.9%	
Plant and machine operators and assemblers	113	7.2	20	1.3%	93	5.9%	33	2.1%	60	3.8%	
Elementary occupations	107	6.8	26	1.7%	81	5.2%	26	1.7%	55	3.5%	
Personnel in government	78	5.0	17	1.1%	61	3.9%	23	1.5%	38	2.4%	
Education level											0.000
Lower than senior secondary	219	14.0	24	1.5%	195	12.5%	26	1.7%	169	10.8%	
Senior secondary	204	13.0	48	3.1%	156	10.0%	45	2.9%	111	7.1%	
Bachelor	988	63.1	188	12.0%	800	51.1%	270	17.2%	530	33.8%	
Master and doctorate	155	9.9	51	3.3%	104	6.6%	41	2.6%	63	4.0%	
Resident area											0.001
Northeast China	168	10.7	45	2.9%	123	7.9%	50	3.2%	73	4.7%	
North China	291	18.6	53	3.4%	238	15.2%	87	5.6%	151	9.6%	
Northwest China	138	8.8	23	1.5%	115	7.3%	32	2.0%	83	5.3%	
Central China	395	25.2	53	3.4%	342	21.8%	74	4.7%	268	17.1%	
South China	318	20.3	85	5.4%	233	14.9%	76	4.9%	157	10.0%	
East China	163	10.4	32	2.0%	131	8.4%	41	2.6%	90	5.7%	
Southwest China	93	5.9	20	1.3%	73	4.7%	22	1.4%	51	3.3%	
Monthly DPI (RMB)											0.000
3000 or below	162	10.3	58	3.7%	104	6.6%	41	2.6%	63	4.0%	
3001–6000	356	22.7	81	5.2%	275	17.6%	106	6.8%	169	10.8%	
6001–10,000	501	32.0	82	5.2%	419	26.8%	130	8.3%	289	18.5%	
10,001–20,000	267	17.0	46	2.9%	221	14.1%	62	4.0%	159	10.2%	
20,001–30,000	101	6.4	30	1.9%	71	4.5%	20	1.3%	51	3.3%	
30,001–40,000	123	7.9	8	0.5%	115	7.3%	6	0.4%	109	7.0%	
40,001 or above	56	3.6	6	0.4%	50	3.2%	17	1.1%	33	2.1%	
Personal health history (multiple answers can be chosen)											
No chronic illness	621	39.7	208	13.3%	413	26.4%	177	11.3%	236	15.1%	0.000
Hypertension	206	13.2	31	2.0%	175	11.2%	40	2.6%	135	8.6%	0.019
Diabetes	167	10.7	26	1.7%	141	9.0%	32	2.0%	109	7.0%	0.034
Heart disease	129	8.2	18	1.1%	111	7.1%	23	1.5%	88	5.6%	0.020
Stroke	192	12.3	14	0.9%	178	11.4%	22	1.4%	156	10.0%	0.000
Gout	208	13.3	6	0.4%	202	12.9%	32	2.0%	170	10.9%	0.000
Lung disease	117	7.5	13	0.8%	104	6.6%	17	1.1%	87	5.6%	0.001
Arthritis	286	18.3	19	1.2%	267	17.0%	64	4.1%	203	13.0%	0.010
Cataracts	32	2.0	4	0.3%	28	1.8%	10	0.6%	18	1.1%	0.539
Neurological disorders	109	7.0	9	0.6%	100	6.4%	20	1.3%	80	5.1%	0.018
Stomach disease	307	19.6	40	2.6%	267	17.0%	81	5.2%	186	11.9%	0.968
Other	49	3.1	8	0.5%	41	2.6%	9	0.6%	32	2.0%	0.230

^a^ A chi-square test was used to compare the two groups (acute pain and chronic pain group).

**Table 2:** Functional impacts of pain on acute and chronic pain sufferers.

	**Total** **(*n* = 1255)**		**Pain Group**				***p*-Value ^a^**
			**Acute Pain (*n* = 381, 24.4%)**		** Chronic Pain (*n* = 873, 55.7%)**		
	**M (SD)**	**Mdn (25%IQR)**	**M (SD)**	**Mdn (25%IQR)**	**M (SD)**	**Mdn (25%IQR)**	
Mood	5.59 (1.40)	6.00 (5.00)	5.29 (1.50)	5.00 (4.00)	5.72 (1.33)	6.00 (5.00)	0.000
Family dynamics	2.21 (0.96)	2.00 (1.00)	2.03 (0.89)	2.00 (1.00)	2.29 (0.97)	2.00 (1.00)	0.000
Social relationship	2.47 (0.89)	3.00 (2.00)	2.18 (0.87)	2.00 (1.00)	2.60 (0.86)	3.00 (2.00)	0.000
Working performance	2.71 (0.89)	3.00 (2.00)	2.50 (0.94)	3.00 (2.00)	2.80 (0.86)	3.00 (2.00)	0.000
Physical activity	2.81 (0,75)	3.00 (2.00)	2.64 (0.80)	3.00 (2.00)	2.89 (0.71)	3.00 (3.00)	0.000
	***n***	**%**	***n***	**%**	***n***	**%**	***p*-value ^b^**
Impact of pain on family dynamics							
Unable to take care of children	307	24.5	83	21.7	224	25.7	0.033
Unable to take care of the elderly	414	33.0	109	28.5	305	34.9	0.019
Family members cannot understand my pain	553	44.1	141	36.9	412	47.2	0.002
Unable to do housework	473	37.7	125	32.7	348	39.9	0.017
Impact of pain on social relationships							
Do not want to leave the house and hang out with friends	437	34.8	126	33.0	311	35.6	0.000
Unwilling to share my suffering with others	599	47.7	143	37.4	456	52.2	0.000
Fear that I must leave due to sudden pain while meeting with others	665	53.0	181	47.4	484	55.4	0.000
Impact of pain on working performance							
Cannot concentrate on my work	655	52.2	209	54.7	446	51.1	0.000
Working procrastination due to frequent hospital visits	614	48.9	148	38.7	466	53.4	0.000
Absenteeism	457	36.4	118	30.9	339	38.8	0.000
Lack of work competence	481	38.3	137	35.9	344	39.4	0.000

^a^ A Mann–Whitney U test was used to compare the pain groups (acute pain and chronic pain group). ^b^ A chi-square test was used to compare the two groups.

**Table 3:** The identified pain treatment used and perceived effectiveness of each treatment.

	**Total** **(*n* = 1255)**		**Pain Group**				***p*-Values ^a^**
			**Acute Pain** **(*n* = 381, 24.4%)**		**Chronic Pain** **(*n* = 873, 55.7%)**		
	***n***	**%**	***n***	** %**	***n***	** %**	
Analgesics used							0.000
Yes	840	66.9	201	52.6	639	73.2	
No	415	33.1	181	47.4	234	26.8	
	***n***	**%**	**M (SD)**	**Mdn (25%IQR)**	**M (SD)**	**Mdn (25%IQR)**	***p*-values ^b^**
Non-pharmacological interventions used							
Bed rest	1184	94.3	1.39 (0.98)	1.00 (1.00)	1.36 (0.91)	1.00 (1.00)	0.698
Massage	1166	92.9	1.57 (0.95)	2.00 (1.00)	1.66 (0.94)	2.00 (1.00)	0.243
Deep breathing	1094	87.2	1.15 (0.97)	1.00 (0.00)	1.20 (0.97)	1.00 (0.00)	0.563
Exercise	1027	81.8	1.20 (0.99)	1.00 (0.00)	1.35 (1.05)	1.00 (0.00)	0.033
Hot compress	1002	79.8	1.47 (0.98)	2.00 (1.00)	1.48 (1.00)	2.00 (1.00)	0.947
Analgesic balm/oil	1019	81.2	1.38 (1.06)	1.00 (0.00)	1.56 (1.06)	2.00 (1.00)	0.027
Recreation	1000	79.7	0.91 (0.96)	1.00 (0.00)	1.13 (1.08)	1.00 (0.00)	0.003
Talking to others	1013	80.7	0.83 (0.86)	1.00 (0.00)	0.92 (0.90)	1.00 (0.00)	0.124
Listening to music	1010	80.5	0.94 (0.98)	1.00 (0.00)	0.93 (1.00)	1.00 (0.00)	0.839
Cold compress	956	76.2	1.04 (0.97)	1.00 (0.00)	1.09 (0.97)	1.00 (0.00)	0.464
Acupuncture	908	72.4	1.09 (1.11)	1.00 (0.00)	1.29 (1.08)	1.00 (0.00)	0.003
Nerve stimulation therapy	843	67.2	0.98 (1.14)	1.00 (0.00)	1.26 (1.09)	1.00 (0.00)	0.000
Aromatherapy	795	63.3	0.71 (0.98)	0.00 (0.00)	0.84 (1.04)	0.00 (0.00)	0.042
Psychotherapy	851	67.8	0.99 (1.06)	1.00 (0.00)	1.21 (1.16)	1.00 (0.00)	0.004

^a^ A chi-square test was used to compare the two groups (acute pain and chronic pain group). ^b^ A Mann–Whitney U test was used to compare the pain groups.

**Table 4:** Care-seeking behaviors in terms of pain management.

	Total (*n* = 1255)		Pain Group				*p*-Value ^a^
			Acute Pain (*n* = 381, 24.4%)		Chronic Pain (*n* = 873, 55.7%)		
	*n*	%	*n*	%	*n*	%	
Sources of pain information acquisition							
No idea	304	24.2	294	77.0	216	24.7	0.516
Websites	618	49.2	201	52.6	417	47.8	0.114
Mobile applications	684	54.5	213	55.8	471	54.0	0.554
Medical staff	592	47.2	182	47.6	410	47.0	0.824
Friends	492	39.2	118	30.9	374	42.8	0.000
Posters	170	13.5	32	8.4	138	15.8	0.000
Newspaper/magazine	104	8.3	23	6.0	81	9.3	0.054
Internet usage for pain-related purposes							
Chronic pain information search	674	53.7	207	54.2	467	53.5	0.820
Pain treatment search	792	63.1	224	58.6	568	65.1	0.030
Therapists	444	35.4	136	35.6	308	35.3	0.913
Entertainment for distraction	429	34.2	123	32.2	306	35.1	0.327
Contacting peer patients	337	26.9	80	20.9	257	29.4	0.002
Contacting support group	233	18.6	32	8.4	201	23.0	0.000
Relaxation training	189	15.1	57	14.9	132	15.1	0.928

^a^ A chi-square test was used to compare the two groups (acute pain and chronic pain groups).

**Table 5:** Participant preferences in terms of pain management education.

	Total (*n* = 1255)		Pain Group ^a^				*p*-Value ^a^
			Acute Pain (*n* = 381, 24.4%)		Chronic Pain (*n* = 873, 55.7%)		
	*n*	%	*n*	%	*n*	%	
Received pain education before							0.000
Yes	39	3.1	4	1.0	35	4.0	
No	1216	96.9	378	99.0	838	96.0	
Willingness to participate in online pain education interventions							0.016
Yes	1172	93.4	347	90.8	825	94.5	
No	83	6.6	35	9.2	48	5.5	
Preferences of pain education methods							
Printed handbook	477	38.0	155	40.6	322	36.9	0.215
On-site education	593	47.3	195	51.0	398	45.6	0.075
Peer support group	804	64.1	211	55.2	593	67.9	0.000
Online education led by intervention deliverer	542	43.2	146	38.2	396	45.4	0.019
Self-management via mobile application	635	50.6	201	52.6	434	49.7	0.344
Preferences of pain education duration							0.000
Up to one week	184	14.7	96	25.1	88	10.1	
2–4 weeks	536	42.7	159	41.6	377	43.2	
5–8 weeks	308	24.5	71	18.6	237	27.1	
More than 2 months	221	17.6	55	14.4	166	19.0	
Other	6	0.5	1	0.3	5	0.6	
Preferences of pain education settings							0.000
30 min, once a week	254	20.2	121	31.7	133	15.2	
1 h, once a week	282	22.5	88	23.0	194	22.2	
30 min, 2–3 times a week	370	29.5	112	29.3	258	29.6	
1 h, 2–3 times a week	230	18.3	37	9.7	193	22.1	
30 min, over 3 times a week	79	6.3	17	4.5	62	7.1	
1 h, over 3 times a week	40	3.2	7	1.8	33	3.8	
Preferences of pain education topic							
Definition and mechanism of pain	482	38.4	174	45.5	308	35.3	0.001
Self-assessment of pain	659	52.5	207	54.2	452	51.8	0.431
Effects of pain	764	60.9	211	55.2	553	63.3	0.007
How acute pain become chronic	630	50.2	207	54.2	423	48.5	0.062
Disease and pain	545	43.4	168	44.0	377	43.2	0.794
Medications and side-effects	717	57.1	199	52.1	518	59.3	0.017
Non-drug treatment	407	32.4	135	35.3	272	31.2	0.145
Preferences of other pain management methods							
Exercise therapy	718	57.2	217	56.8	501	57.4	0.848
Psychotherapy	783	62.4	208	54.5	575	65.9	0.000
Game therapy	461	36.7	134	35.1	327	37.5	0.421
Massage therapy	608	48.4	189	49.5	419	48.0	0.629

^a^ A chi-square test was used to compare the groups.

## Data Availability

The data that support the findings of this study are available from the corresponding author upon reasonable request. The data generated during and/or analyzed during the current study are not publicly accessible in order to uphold the privacy assurances made to participants. However, they can be obtained from the corresponding author upon reasonable request.
